# Renal Effects of Energy Drink Consumption: A Systematic Review and Meta-Analysis of Preclinical Studies

**DOI:** 10.3390/toxics14050376

**Published:** 2026-04-28

**Authors:** Alfredo G. Casanova, Ana I. Morales, Laura Vicente Vicente

**Affiliations:** 1 Unidad de Toxicología, Departamento de Fisiología y Farmacología, Universidad de Salamanca (USAL), 37007 Salamanca, Spain; alfredogcp@usal.es (A.G.C.); amorales@usal.es (A.I.M.); 2 Instituto de Investigación Biomédica de Salamanca (IBSAL), 37007 Salamanca, Spain; 3 Translational Research on Renal and Cardiovascular Diseases (TRECARD), 37007 Salamanca, Spain

**Keywords:** energy drink, kidney injury, consumption, caffeine, food safety

## Abstract

In recent decades, there has been a worrying increase in the consumption of energy drinks, especially among the young population. These beverages have been linked to effects primarily on the nervous and cardiovascular systems; however, renal consequences remain poorly understood, particularly those associated with long-term consumption. The heterogeneity in consumption patterns, together with variability in consumer profiles, has limited the ability to obtain conclusive and consistent evidence on this issue. Therefore, it is essential to address this topic from a preclinical perspective under controlled experimental conditions. The aim of the present study was to evaluate the available preclinical evidence through a systematic review followed by a meta-analysis. Nineteen studies met the inclusion criteria, with rats being the most commonly used experimental model. Overall, the results show a significant association between energy drink consumption and impaired kidney function, as reflected in elevated levels of serum biomarkers such as creatinine, urea, and uric acid. Among these, serum creatinine (mean Hedges’ g = −1.40; 95% confidence interval: −2.55 to −0.25) and urea (Hedges’ g = −1.93; 95% confidence interval: −2.99 to −0.87) had the highest effect sizes and greater statistical robustness, suggesting a particularly significant impact on kidney function. Although fewer studies reported increased uric acid levels, this parameter may have pathophysiological relevance. Elevated uric acid has been associated with mechanisms such as inflammation, oxidative stress, and endothelial dysfunction, which may contribute to the progression of renal damage. Additionally, morphological alterations in glomerular and tubular structures were observed, particularly after prolonged exposure and at high doses. Despite the need for methodological improvements in future research, these findings highlight potential adverse renal effects that should be considered in the development of regulatory policies on energy drink consumption.

## 1. Introduction

In recent years, the consumption of energy drinks (EDs) has experienced a worrying increase worldwide. According to the European Food Safety Authority report issued in 2013, 68% of European adolescents (aged 10–18) had consumed EDs at least once in their lifetime, while in the adult group (18–65 years old), this number decreased to 30%. Alarmingly, around 18% of children aged between 3 and 10 also reported having consumed these beverages, highlighting a growing trend among youngsters, who are more susceptible to the effects of these stimulants [1].

This consumption pattern is not limited to Europe but it is also reflected on other continents. A recent global analysis, based on a meta-analysis by Aonso-Diego et al. [2] has confirmed that ED consumption remains high worldwide, especially among adolescent and young adult populations. This study posits that, despite concerted efforts to disseminate information regarding health risks associated with excessive consumption of these beverages, demand continues to increase [2]. This phenomenon is reflected in the projected growth of the global ED market, which is expected to continue to expand in the coming years [3]. Some populations have been identified as having a marked increase in ED consumption, such as gamers [4] and sports people [5], who turn to these drinks primarily for their ability to improve physical and mental performance. These groups benefit from the ingredients present in EDs, such as caffeine (50–500 mg per serving), taurine (1000–2000 mg), glucuronolactone (600–1200 mg), guarana (which provides additional caffeine), ginseng (200–400 mg), and sugars (20–40 g), among other compounds [6]. The primary effects sought by ED consumers appear to be associated with their high caffeine content. Caffeine has been demonstrated to exert a positive effect on several domains, including cognitive performance, visuospatial ability, and mood. Moreover, it has been demonstrated that caffeine has a stimulant effect, promoting alertness, concentration, and short-term memory [4,7]. Evidence has demonstrated that caffeine can enhance sporting performance. Consuming an ED containing a minimum of 3 mg/kg of caffeine before exercise can improve performance in endurance events such as cycling and 5 km races, increase distance covered at high intensity in team sports such as football and rugby, and improve time in sprint events such as 50 m swimming. It has also been demonstrated that caffeine has the capacity to enhance reaction and response times in specific circumstances [5,8]. Although caffeine is the main ingredient in EDs, other stimulants such as taurine may also play a role in the effects sought by consumers.

These ingredients can cause adverse effects, the most common being an increase in blood pressure and heart rate, which can increase the risk of cardiovascular events in susceptible individuals [9]. In addition, a prolongation of the QTc interval after consumption has been reported, which may predispose consumers to cardiac arrhythmias [10]. Excessive consumption of EDs has been demonstrated to induce insomnia, anxiety, and depressive symptoms [9,11] and even withdrawal symptoms when consumption is stopped [12]. Liver damage has also been documented [13,14], with some severe cases requiring transplantation [15]. However, the renal effects of EDs remain poorly understood. Although isolated cases of acute renal damage have been reported, these correspond to extreme situations such as excessive consumption (e.g., 12 cans of 250 mL in a single day) [16] or the presence of pre-existing pathologies such as cancer [17] or diabetes [18], which could be considered as renal risk factors. Although these events suggest a possible association between EDs and renal damage, further investigation is required to substantiate this association. The absence of consistent clinical evidence underscores the pressing need for further research in this domain. In addition to being scarce, the available clinical data is subject to many biases in terms of the exposed population, doses, consumption duration, and the concomitant administration of other potentially nephrotoxic substances and/or comorbidities. In this context, preclinical research provides a controlled environment in which the relationship between energy drinks and renal impairment can be established with greater precision, while minimizing the variability present in clinical studies. This work therefore aims to analyze the available preclinical evidence in this field through a systematic review followed by a meta-analysis.

## 2. Materials and Methods

The entire procedure described below was carried out in accordance with the Preferred Reporting Items for Systematic Reviews and Meta-Analyses guidelines. The protocol of this systematic review was registered on the PROSPERO platform (code: CRD42025629561, date: 12 February 2025).

### 2.1. Article Search

**Figure 1:**
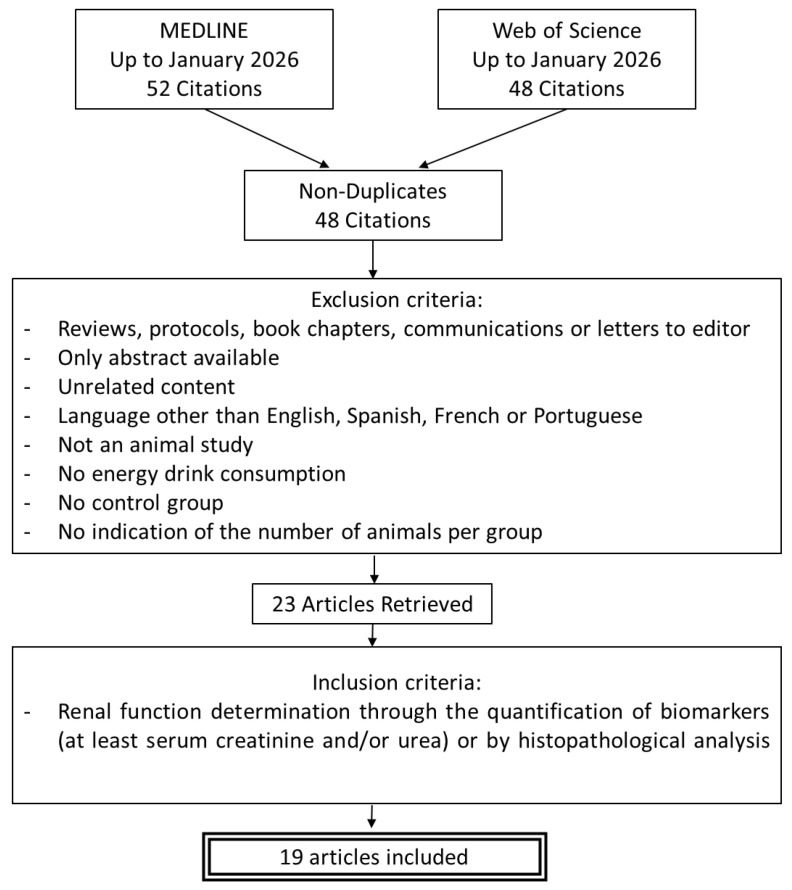
Flowchart of the search process.

The complete paper search and selection process is shown in Figure 1. The literature search was performed for scientific articles related to possible kidney damage caused by energy drinks included in the Medline and Web of Science databases up to January 2025 (last search date: 8 January 2026). The following search strategy was used (MeSH terms and keywords were used in order to maximize article mining): on the PUBMED platform, the MeSH terms used were “energy drinks/adverse effects” [MeSH]. On both platforms, the terms were used independently as follows: “Energy drinks” AND (kidney OR renal); Caffeine AND taurine AND (kidney OR renal) AND (injury OR damage).

### 2.2. Exclusion and Inclusion Criteria

Any article that met at least one of the following criteria was excluded: (1) reviews, protocols, book chapters, communications, or letters to editor; (2) only abstract available; (3) unrelated content; (4) language other than English, Spanish, French, or Portuguese; (5) not an animal study; (6) no ED consumption; (7) no control group; (8) no indication of the number of animals per group. Of the remainder, only those that evaluated renal damage through the quantification of biomarkers (at least serum creatinine and/or urea) or through histopathological analysis were included. These selection stages were carried out independently by two researchers (A.G.C. and L.V.V.). If there were discrepancies, a third researcher (A.I.M.) resolved them.

### 2.3. Data Extraction

The following data were extracted from each article: study identification (first author and year), species, treatment administered (ED, dose, and time), size of study groups, renal function biomarkers (where available), and histopathological study findings (where available).

Once the profile of biomarkers used in most of the studies had been examined, serum creatinine, urea, and uric acid were selected for the meta-analysis stage. The mean value and standard deviation (SD) of these biomarkers were recorded for the ED-treated group and the control group. To ensure the comparability of these data, when the original article did not provide serum concentrations in mg/dL, the corresponding numerical conversion was performed. The SD was extracted directly from the work or calculated from the provided dispersion parameters.

### 2.4. Risk of Bias Assessment

The risk of bias of the included animal studies was assessed using the SYRCLE’s risk of bias tool for animal studies [19]. This tool is an adapted version of the Cochrane risk of bias tool designed specifically for preclinical in vivo research. It evaluates 10 domains of bias, including selection, performance, detection, attrition, reporting, and other sources of bias. Each domain was judged as having low, high, or unclear risk of bias based on predefined signaling questions. The assessment was performed by critically reviewing the methodological information reported in each study, including randomization procedures, allocation concealment, blinding, outcome assessment, and completeness of outcome data.

### 2.5. Meta-Analysis

Heterogeneity between studies was assessed using the chi-squared Q-test under the null hypothesis of homogeneity (*p* < 0.05 indicates heterogeneity) and the I^2^ parameter (I^2^ > 50% indicates high heterogeneity). Since the studies were heterogeneous for the three biomarkers, the random effects model was applied for the meta-analysis of the data. Based on the difference in means between the control group and the treated group in each study (that is, based on the result of the following subtraction: the mean biomarker level in the control group minus the mean biomarker level in the treated group), Hedges’ g (a standardized measure of effect size that is particularly suitable when sample sizes are small) and its 95% confidence interval were calculated for each study, and the combined value of these parameters was calculated [20]. Forest plots were constructed from these parameters.

In addition, funnel plots were constructed showing the Hedges’ g of each study versus its standard error to assess potential publication bias. Begg and Mazumdar [21] and Egger et al. [22] asymmetry tests were also applied.

All analyses described in this section were performed using the Meta-Essentials set of workbooks [23].

## 3. Results

### 3.1. Description of Included Studies

The characteristics of the studies included in this work are presented in Table 1.

Following the completion of the search and screening phase, a total of 19 preclinical articles were included in the review. These articles evaluated the effect of different doses and brands of EDs on animals, with the exception of one article which evaluated the effect on rabbits. Treatment duration varied considerably, from 15 days of exposure to 120 days. Ten of them assessed animal renal function using serum creatinine and urea levels, followed by serum uric acid (quantified in six studies) and calcium, phosphorus, sodium, and potassium (considered in four or fewer studies). Fifteen of the included studies reported a renal histopathological study, but generally did not show quantitative data, so the meta-analysis was limited to studies that provided biomarker numerical data.

The risk of bias assessment using SYRCLE’s tool (Supplementary Table S1) showed that most of the included studies were rated as having an unclear risk of bias across several domains. This was mainly due to insufficient reporting of methodological details such as randomization procedures, allocation concealment, and blinding of investigators and outcome assessors, rather than evidence of inadequate conduct of these procedures. A smaller number of studies were judged to have a low risk of bias, particularly in domains where randomization or baseline characteristics were explicitly described. In many cases, studies reported approval by an ethical or animal care committee, where more detailed methodological information may have been specified, although this was not fully reported in the published articles. Overall, the predominance of unclear risk of bias reflects limited methodological transparency in the reporting of animal studies rather than necessarily poor methodological quality.

### 3.2. Evaluation of Renal Function

**Figure 2:**
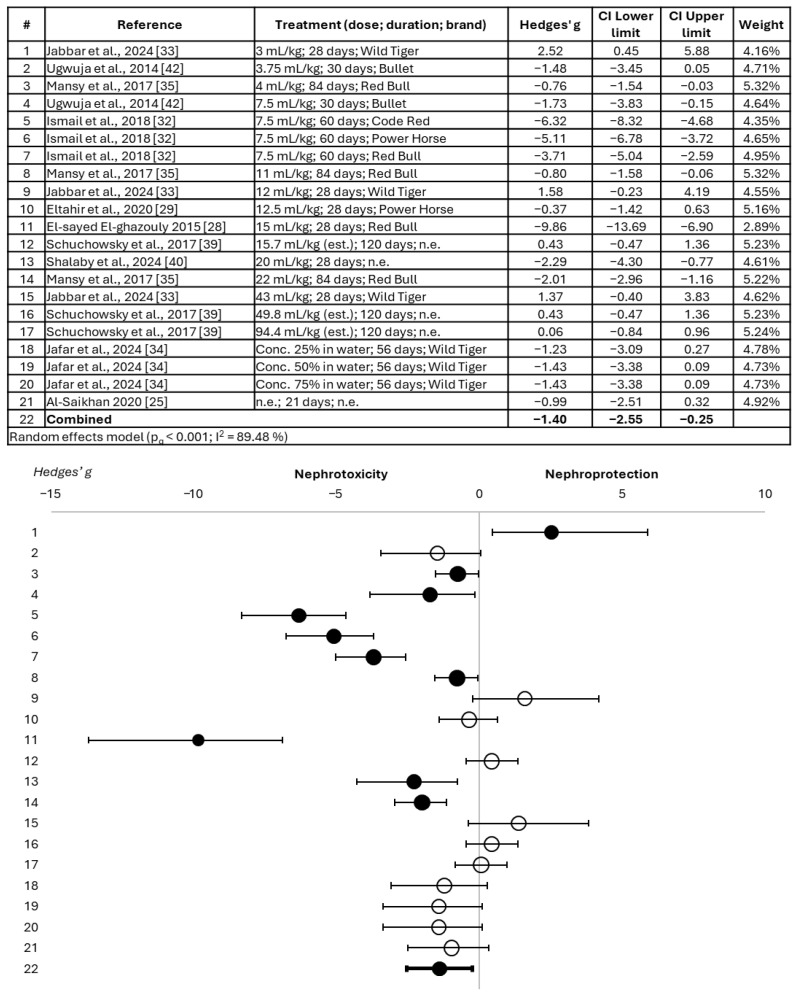
Forest plot showing the alteration of serum creatinine in animals treated with energy drinks versus those belonging to the control group [25,28,29,32,33,34,35,39,40,42]. Effect size is measured as Hedges’ g ± 95% CI. The black circles represent significant effects; the white circles represent non-significant effects. CI: confidence interval; est.: estimated; n.e.: not specified.

The forest plot presenting the Hedges’ g parameter (individual for each study and combined) for serum creatinine is shown in Figure 2.

It is observed that 9 of the 21 treatments tested proved to be significantly nephrotoxic based on serum creatinine, obtaining a combined value also statistically significant in favor of nephrotoxicity (Hedges’ g mean = −1.40; 95% confidence interval = −2.55, −0.25). No clear dose–toxicity relationship was observed *a priori*, nor was a more pronounced nephrotoxic effect noted with prolonged administration. Only one study showed a more harmful effect, in which the Power Horse drink was administered at a dose of 12.5 mL/kg for 28 days. However, this appears to be peculiar to this single study, as other studies with a similar treatment did not show the same change in serum creatinine.

**Figure 3:**
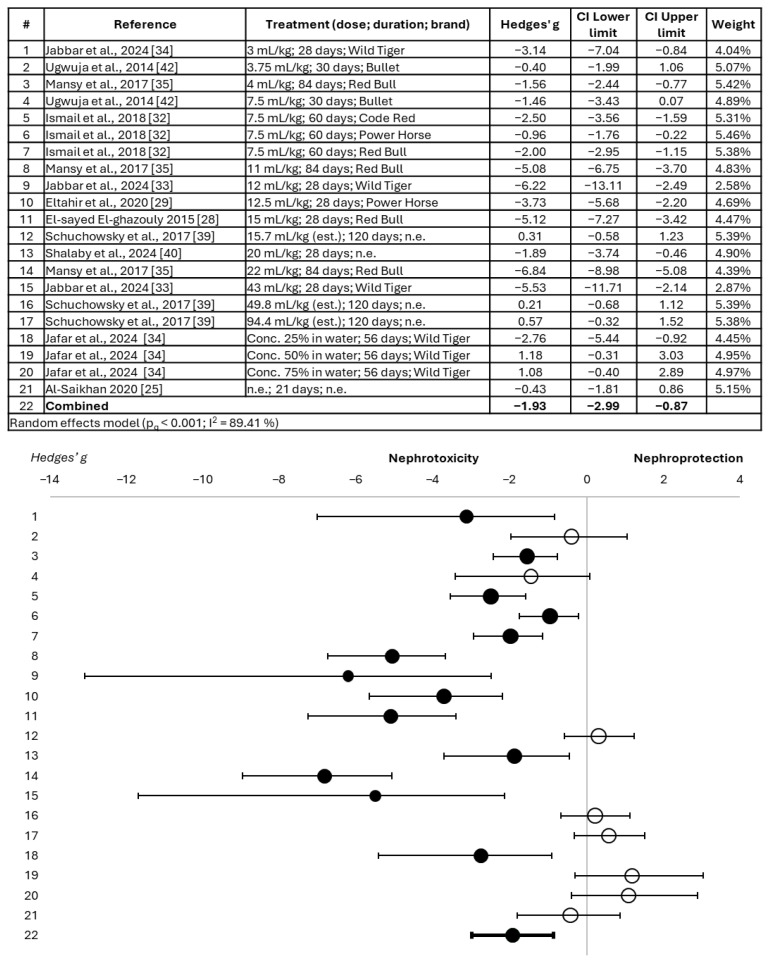
Forest plot showing the alteration of serum urea in animals treated with energy drinks versus those belonging to the control group [25,28,29,32,33,34,35,39,40,42]. Effect size is measured as Hedges’ g ± 95% CI. The black circles represent significant effects; the white circles represent non-significant effects. CI: confidence interval; est.: estimated; n.e.: not specified.

**Figure 4:**
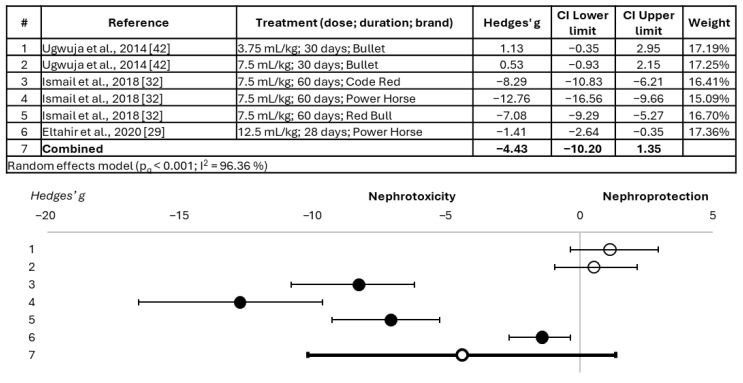
Forest plot showing the alteration of serum uric acid in animals treated with energy drinks versus those belonging to the control group [29,32,42]. Effect size is measured as Hedges’ g ± 95% CI. The black circles represent significant effects; the white circles represent non-significant effects. CI: confidence interval; est.: estimated; n.e.: not specified.

The nephrotoxic effect of the tested EDs is more evident in the analysis of serum urea modification (Figure 3), which showed a significant combined Hedges’ g of −1.93 (95% confidence interval = −2.99; −0.87). For this parameter, 13 of the 21 experimental treatments evaluated showed statistically significant detrimental effects on the kidneys. No administration pattern was identified as being more toxic, nor was a dose–duration–effect relationship identified. Finally, this meta-analysis revealed that, although serum uric acid alteration (Figure 4) showed an evident tendency towards nephrotoxicity, there is not yet sufficient scientific evidence to qualify it as significant. However, four of the six treatments showed a significant nephrotoxic effect for this parameter. Again, the Power Horse drink (administered at a dose of 7.5 mL/kg for 60 days) showed the most detrimental effects for this biomarker.

### 3.3. Publication Bias Evaluation

**Figure 5:**
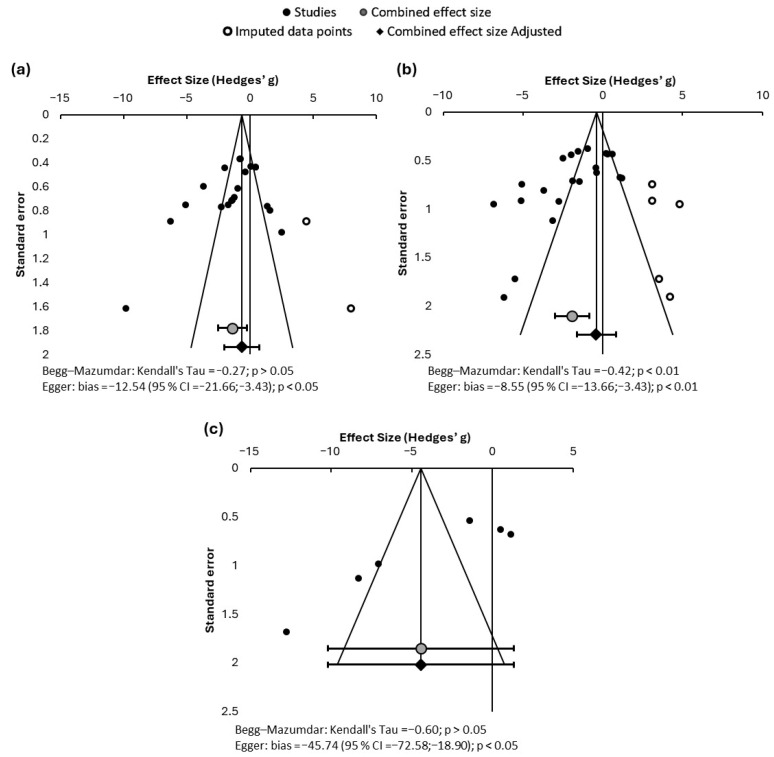
Funnel plots and asymmetry tests for serum creatinine (**a**), urea (**b**), and uric acid (**c**). Effect size is measured as Hedges’ g ± 95% confidence interval.

The graphical analysis and the results of the asymmetry tests applied are shown in Figure 5. For the three parameters analyzed, some asymmetry was identified, both graphically and numerically (although the test results sometimes contradict each other). It is therefore possible that publication bias exists. However, when analyzing the asymmetry shown in the funnel plots (which reveal a gap in published studies reporting highly nephroprotective effects attributable to ED), it is possible that this is due not so much to a true publication bias, but rather due to the fact that nephroprotection is not an effect that has been attributed to these products to date, which explains why it is more common to find publications showing no effect (neither harmful nor beneficial) or showing nephrotoxic effects.

### 3.4. Histopathological Analysis

To understand the possible alterations in renal morphology caused by the consumption of ED, the information reported in the fifteen articles that showed renal histology studies was analyzed (Table 2). These results suggest that chronic consumption of ED can induce renal damage, affecting both glomerular and tubular structure. However, determining a dose-dependent relationship is complex. When comparing across studies, no consistent pattern emerges, with some investigations reporting no adverse effects even at high doses [40], while others show histological alterations even at lower doses [26,27]. However, in studies where multiple doses were assessed simultaneously, more pronounced renal damage is observed in groups exposed to higher doses, suggesting a possible dose–response relationship [35,36,37,41].

## 4. Discussion

The increasing ED consumption has been linked to several health conditions, with renal impairment emerging as a potential consequence of long-term use. However, the renal effects remain poorly understood. To the best of our knowledge, this study is the first systematic review and meta-analysis addressing ED-induced renal injury. Despite the bias of extrapolation to humans, the results obtained in animals provide us with information on the subject. However, it should be noted that these results do not include all the factors associated with human consumption, such as pathologies, individual vulnerability and interactions.

The findings of this study show an association between ED consumption and renal alterations, as evidenced by increased blood creatinine, urea and uric acid levels, as well as morphological changes to the glomerular and tubular structures. The authors selected creatinine and urea as the biomarkers for assessing renal function because these are commonly used in clinical practice [43]. A limited number of studies have also documented an increase in uric acid levels. Although this is not a marker commonly associated with the diagnosis of kidney damage, elevated uric acid levels are thought to contribute to kidney damage through mechanisms such as inflammation, oxidative stress and endothelial dysfunction [44]. Consequently, in addition to its potential value as a biomarker, uric acid might also act as a contributing factor in the progression of renal damage.

Despite the apparent impact of ED on renal function, no clear dose–response or exposure time-response relationship is observed when a meta-analysis of biomarker data is conducted. This lack of correlation is further supported by histological findings. However, when studies are evaluated individually, a relationship between higher doses and greater damage appears to be evident in those that assess multiple dose levels [35,36,37,41]. This may be partly due to the use of different rat strains with varying sensitivity to the renal effects of these beverages or to an inability to detect a consistent pattern. To resolve this dilemma, it is essential to enhance experimental design, as current studies often simplify the assessment of renal function, relying predominantly on biomarkers that have several limitations. These traditional markers, such as serum creatinine and urea, are not sensitive or specific enough to detect early renal damage. In contrast, histological findings reveal alterations consistent with chronic kidney disease in its early stages, that may not be detected using these standard biomarkers. This suggests that current methodologies may fail to identify subtle renal impairments before they progress. Fortunately, new biomarkers are emerging that offer greater sensitivity and specificity than conventional serum creatinine and urea. These biomarkers could play a crucial role in improving early diagnosis and monitoring in clinical settings by providing a more accurate and timely assessment of renal health and detecting kidney damage at earlier stages. For example, to determine tubular function, urinary excretion of proteins as neutrophil gelatinase-associated lipocalin (NGAL), kidney injury molecule-1 (KIM-1), N-acetyl-β-D-glucosaminidase (NAG), and liver-type fatty acid binding protein (L-FABP) can be evaluated; to determine whether inflammatory or tissue repair processes have occurred, interleukin-18 (IL-18), monocyte chemotactic protein-1 (MCP-1), osteopontin (OPN), a chemokine ligand with CC motif 14 (CCL14), among others, can be tested [45]. These biomarkers would provide more information on the type of damage, which, combined with histological studies, would help to better understand how ED affects kidney function and to distinguish between acute, chronic and sub-chronic processes.

In this study, it is observed that concerns about the renal effects of ED consumption are primarily focused on long-term exposure, rather than acute intake. This may be because acute effects are more evident and have already been reported in clinical settings. For instance, some case reports have described acute kidney injury following excessive consumption of ED, particularly when combined with factors such as intense physical activity, dehydration, or the concurrent alcohol consumption [18,46]. In contrast, the potential effects of chronic ED consumption on kidney health remain less clearly defined in humans. Two clinical studies have reported that the regular consumption of 2 to 7 cans of ED per week is associated with slight increases in serum creatinine levels, although these changes are less pronounced than those observed in preclinical models [47,48]. Conversely, a larger cross-sectional study involving 1031 participants revealed a higher prevalence of kidney stone formation among regular ED consumers, though no significant changes in kidney function biomarkers were detected [49]. A key challenge in obtaining conclusive evidence from human studies is the difficulty in accurately quantifying both the dose and duration of ED consumption. A significant proportion of the available data relies on retrospective reports, which limits their reliability. Therefore, well-designed prospective longitudinal studies are needed to evaluate the long-term progression of renal function in ED consumers, as opposed to assessments based solely on cross-sectional data collected at a single time point. Furthermore, the majority of these studies have been conducted on the adult population. However, the current focus is on the pediatric population, as it is estimated that 9% of cases of acute tubulointerstitial nephritis are associated with the use of ED [50]. Although the results observed in preclinical studies cannot be easily extrapolated to humans, the nephritis detected in children could be associated with the presence of inflammatory infiltrates, as well as evidence of fibrosis observed in the histological findings.

Of all the ED components, the high caffeine content appears to be the main cause of renal damage, via various mechanisms of action such as increased diuresis and oxidative stress. It has been demonstrated that caffeine exerts an effect on renal autoregulation and results in a reduction in renal blood flow [51]. Furthermore, the combined effects of caffeine, taurine and high sugar levels can result in structural changes to renal tissue, including edema and interstitial inflammation [41].

## 5. Conclusions

The results of this meta-analysis suggest that long-term consumption of EDs leads to renal damage, affecting both kidney morphology and function. However, the current evidence base is insufficient to ascertain whether this effect is dose- or time-dependent. Further studies incorporating design improvements are needed to address this uncertainty. The generation of this evidence is of crucial importance to inform public health policies aimed at regulating ED consumption, particularly among vulnerable populations such as children and adolescents.

## Figures and Tables

**Table 1:** Descriptive characteristics of the included studies. ED: energy drink; est.: estimated; n.i.: not indicated.

Study Identification	Animal Species	ED Brand	ED Dose	ED Time of Administration	ED Group Size	Serum Biomarkers of Nephrotoxicity							Histology
						Creatinine	Urea	Uric Acid	Ca	P	K	Na	
Al-Basher et al., 2018 [24]	Swiss Webster mice (pregnant)	Red Bull	2.5 mL/kg	From the first day of pregnancy until the 15th day after birth	5								x
			5 mL/kg	From the first day of pregnancy until the 15th day after birth	5								x
Al-Saikhan, 2020 [25]	Wistar rats	n.i.	n.i.	21 days	5	x	x						x
Bano et al., 2020 [26]	Sprague Dawley rats	Red Bull	3.57 mL/kg	28 days	10								x
			3.57 mL/kg	56 days	10								x
Bano et al., 2020 [27]	Sprague Dawley rats	Red Bull	3.57 mL/kg	28 days	10	x							x
			3.57 mL/kg	56 days	10	x							x
El-sayed El-ghazouly, 2015 [28]	Albino rats	Red Bull	15 mL/kg	28 days	10	x	x						x
Eltahir et al., 2020 [29]	Albino rats	Power Horse	12.5 mL/kg	28 days	8	x	x	x					x
Hanna et al., 2024 [30]	Wistar rats (pregnant)	n.i.	5 mL/kg	15 days	6								x
			10 mL/kg	15 days	6								x
Hegazy et al., 2022 [31]	Albino rats	Red Bull	22 mL/kg	56 days	10								x
Ismail et al., 2018 [32]	Wistar rats	Red Bull	7.5 mL/kg	60 days	12	x	x	x	x	x			x
		Code Red	7.5 mL/kg	60 days	12	x	x	x	x	x			x
		Power Horse	7.5 mL/kg	60 days	12	x	x	x	x	x			x
Jabbar et al., 2024 [33]	Wistar rats	Wild Tiger	3 mL/kg	28 days	3	x	x						
			12 mL/kg	28 days	3	x	x						
			43 mL/kg	28 days	3	x	x						
Jafar et al., 2024 [34]	Wistar rats	Wild Tiger	25% Tiger + 75% water	56 days	4	x	x						
			50% Tiger + 50% water	56 days	4	x	x						
			75% Tiger + 25% water	56 days	4	x	x						
Mansy et al., 2017 [35]	Sprague Dawley rats	Red Bull	4 mL/kg	84 days	15	x	x						x
			11 mL/kg	84 days	15	x	x						x
			22 mL/kg	84 days	15	x	x						x
Memudu et al., 2020 [36]	Wistar rats	Power Horse	2 mL/kg	21 days	5								x
			4 mL/kg	21 days	5								x
Qassim et al., 2022 [37]	Albino rats	Red Bull	10 mL/kg	30 days	10								x
			20 mL/kg	30 days	10								x
Rasheed et al., 2021 [38]	Albino rats	n.i.	15 mL/kg	59 days	30								x
			22 mL/kg	59 days	30								x
Schuchowsky et al., 2017 [39]	Wistar rats	n.i.	15.7 mL/kg (est.)	120 days	10	x	x						
			49.8 mL/kg (est.)	120 days	10	x	x						
			94.4 mL/kg (est.)	120 days	10	x	x						
Shalaby et al., 2024 [40]	Rats	n.i.	20 mL/day	28 days	5	x	x						x
Salih et al., 2018 [41]	Albino rabbits	Red Bull	5 mL/kg (est.)	30 days	10								x
			10 mL/kg (est.)	30 days	10								x
Ugwuja et al., 2014 [42]	Wistar rats	Bullet	3.75 mL/kg	30 days	4	x	x	x					
			7.5 mL/kg	30 days	4	x	x	x	x		x	x	

**Table 2:** Histopathological alterations due to energy drink consumption. DCT: distal convoluted tubule; PCT: proximal convoluted tubule; est.: estimated; n.i.: not indicated creatinine and urea levels, followed by serum uric acid (quantified in six studies) and calcium, phosphorus, sodium, and potassium (considered in four or fewer studies). Fifteen of the included studies reported a renal histopathological study, but generally did not show quantitative data, so the meta-analysis was limited to studies that provided biomarker numerical data.

Autor	Species	Energy Drink (Dose, Time, Brand)	Histological Manifestations
Al-Basher et al., 2018 [24]	Swiss Webster mice (pregnant)	2.5 or 5 mL/kg; from the first day of pregnancy until the 15th day after birth; Red Bull	Exposed newborn mice showed degenerated developing glomeruli and dilated urinary spaces at both doses.
Al-Saikhan 2020 [25]	Wistar rats	n.i.; 21 days; n.e.	No pathological manifestations.
Bano et al., 2020 [26]	Sprague Dawley rats	3.57 mL/kg; 56 or 28 days; Red Bull	Loss of brush border in PCT, necrosis. Severe hemorrhage in renal cortex.
Bano et al., 2020 [27]	Sprague Dawley rats	3.57 mL/kg; 56 or 28 days; Red Bull	Shrinkage of glomerulus and widening of bowman space at both administration durations.
El-sayed El-ghazouly 2015 [28]	Albino rats	15 mL/kg; 28 days; Red Bull	Dilated and congested glomerular capillaries; marked glomerular degeneration with widening of Bowman’s space. Marked distortion and dilatation of renal tubules with cytoplasmic vacuoles and pyknotic nuclei in the lining cells. Some tubules showed loss of their nuclei or sloughed necrotic cells within their lumens. Empty spaces within the renal cortex, dilated and congested blood vessels, and areas of hemorrhage in the cortex.
Eltahir et al., 2020 [29]	Albino rats	12.5 mL/kg; 28 days; Power Horse	No pathological manifestations
Hanna et al., 2024 [30]	Wistar rats (pregnant)	5 or 10 mL/kg; 15 days; n.i.	**Low-Dose Group:** Variation in glomerular size and shape, with some glomeruli shrinking (glomerular tufts). Many glomeruli were fragmented and congested; high dose: enlarged glomeruli and large capsular space. Some glomerular tufts were vacuolated and shrunken; necrosis of total or partial nuclei in DCT and PCT; other cells contain vacuoles. Invasion of inflammatory cells into the injured tubules and corpuscles of the kidney and cellular debris. **High-Dose Group:** A few tubular epithelial cells in the convoluted tubules. Moderate lymphovascular invasion and intratubular mononuclear cell infiltration were seen in the vicinity of blood vessels. Congestion was also observed. Renal tubules had disrupted epithelial lining and degraded cells. The lumen of several distal tubules was dilated.
Hegazy et al., 2022 [31]	Albino rats	22 mL/kg; 56 days; Red Bull	Some glomeruli were sclerotic or shrunken, or segmented or hypercellular, with the narrowing or focal obliteration of the capsular space. Widening of Bowman’s space. Degeneration of PCT lining cells with cytoplasmic vacuolation and cellular debris in their lumen and many disorganized tubules with dilated lumen. Mononuclear cell infiltration.
Ismail et al., 2018 [32]	Wistar rats	7.5 mL/kg; 60 days; Code Red 7.5 mL/kg; 60 days; Power Horse 7.5 mL/kg; 60 days; Red Bull	**Code Red:** Congestion of periglomerular blood vessels. **Power Horse:** Eosinophilic casts inside tubular lumina. **Red Bull:** Moderate hydropic degeneration of tubular epithelium.
Mansy et al., 2017 [35]	Sprague Dawley rats	4 or 11 or 22 mL/kg; 84 days; Red Bull	**High-Dose Group:** Markedly shrunken glomerulus. Renal corpuscle surrounded by incipient fibrosis. Remains of destroyed tubules, signs of degeneration, necrosis, and loss of cell borders. Marked dilatation and congestion of the veins and severe interstitial inflammation.
Memudu et al., 2020 [36]	Wistar rats	2 or 4 mL/kg; 21 days; Power Horse	**High-Dose Group:** Increase in the Bowman’s space, reduction in glomerular cytoarchitecture. Reduced proximal convoluted tubule integrity and surrounding epithelium. **Both Doses:** Slight distortion in histoarchitecture of the kidney under low doses. Severe loss of cell membrane, palely stained cells, and loss of mucin granules.
Qassim et al., 2022 [37]	Albino rats	10 or 20 mL/kg; 30 days; Red Bull	**Low-Dose Group:** Vascular congestion in the glomeruli, as well as in the interstitial space, atrophic glomeruli. Vacuolar degeneration of renal tubular epithelium and coagulative necrosis of renal tubular epithelium. **High-Dose Group:** More atrophic glomeruli than in the low-dose group. Massive coagulative necrosis of renal tubular epithelium.
Rasheed et al., 2021 [38]	Albino rats	15 or 22 mL/kg; 59 days; n.i.	**Both Doses:** Mild perinuclear vacuolization in epithelial cells of renal tubules.
Salih et al., 2018 [41]	Albino rabbits	5 or 10 mL/kg (est.); 30 days; Red Bull	**High-Dose Group:** Glomerular capillary segmentation or lobulation. **Both Doses**: Glomerular swelling. Renal vascular congestion, hemorrhage of interstitial tissue, focal atrophy, and degeneration of the lining epithelium of the DCT and PCT.
Shalaby et al., 2024 [40]	Rats	20 mL/kg; 28 days; n.i.	No pathological manifestations.

## Data Availability

No new data were created in this study. Data sharing is not applicable to this article.

## Supplementary Materials

The following supporting information can be downloaded at: https://www.mdpi.com/article/10.3390/toxics14050376/s1, Table S1: Risk of bias assessment of the included animal studies using the SYRCLE’s tool.

## Abbreviations

The following abbreviations are used in this manuscript:

ED	energy drink
