# Supplementary material for: Renal Effects of Energy Drink Consumption: A Systematic Review and Meta-Analysis of Preclinical Studies
Source: Toxics. 2026 Apr 28;14(5):376. doi: 10.3390/toxics14050376 (PMC13210930; doi:10.3390/toxics14050376)
Supplement: Supplementary file 1 [file toxics-14-00376-s001.zip › toxics-4225697-supplementary.pdf]

Study identification	Sequence generation	Baseline characteristics	Allocation concelament	Random housing	Blinding	Random outcome assessment	Blinding	Incomplete outcome data	Selective outcome reporting	Other sources of bias
Al-Basher et al., 2018 [24]	Unclear risk	Unclear risk	Low risk	Unclear risk	Unclear risk	Unclear risk	Unclear risk	Low risk	Low risk	Unclear risk
Al-Saikhan, 2020 [25]	Unclear risk	Unclear risk	Unclear risk	Unclear risk	Unclear risk	Unclear risk	Unclear risk	Low risk	Low risk	Unclear risk
Bano et al., 2020 [26]	Low risk	Low risk	Unclear risk	Unclear risk	Unclear risk	Unclear risk	Unclear risk	Low risk	Low risk	Unclear risk
Bano et al., 2020b [27]	Low risk	Low risk	Unclear risk	Unclear risk	Unclear risk	Unclear risk	Unclear risk	Low risk	Low risk	Unclear risk
El-sayed El-ghazouly, 2015 [28]	Unclear risk	Unclear risk	Unclear risk	Unclear risk	Unclear risk	Unclear risk	Unclear risk	Low risk	Low risk	Unclear risk
Eltahir et al., 2020 [29]		Unclear risk	Unclear risk	Unclear risk	Unclear risk	Unclear risk	Unclear risk	Low risk	Low risk	Unclear risk
Hanna et al., 2024 [30]	Unclear risk	Unclear risk	Unclear risk	Unclear risk	Unclear risk	Unclear risk	Unclear risk	Low risk	Low risk	Unclear risk
Hegazy et al., 2022 [31]	Unclear risk	Unclear risk	Unclear risk	Unclear risk	Unclear risk	Unclear risk	Unclear risk	Low risk	Low risk	Unclear risk
Ismail et al., 2018 [32]	Unclear risk	Unclear risk	Unclear risk	Unclear risk	Unclear risk	Unclear risk	Unclear risk	Low risk	Low risk	Unclear risk
Jabbar et al., 2024 [33]	Unclear risk	Unclear risk	Unclear risk	Unclear risk	Unclear risk	Unclear risk	Unclear risk	Low risk	Low risk	Unclear risk
Jafar et al., 2024 [34]	Unclear risk	Unclear risk	Unclear risk	Unclear risk	Unclear risk	Unclear risk	Unclear risk	Low risk	Low risk	Unclear risk
Mansy et al., 2017 [35]	Low risk	Low risk	Unclear risk	Unclear risk	Unclear risk	Unclear risk	Unclear risk	Low risk	Low risk	Unclear risk
Memudu et al., 2020 [36]	Unclear risk	Low risk	Unclear risk	Unclear risk	Unclear risk	Unclear risk	Unclear risk	Low risk	Low risk	Unclear risk
Qassim et al., 2022 [37]	Unclear risk	Unclear risk	Unclear risk	Unclear risk	Unclear risk	Unclear risk	Unclear risk	Low risk	Low risk	Unclear risk
Rasheed et al., 2021 [38]	Unclear risk	Low risk	Unclear risk	Unclear risk	Unclear risk	Unclear risk	Unclear risk	Low risk	Low risk	Unclear risk
Schuchowsky et al., 2017 [39]	Unclear risk	Unclear risk	Unclear risk	Unclear risk	Unclear risk	Unclear risk	Unclear risk	Low risk	Low risk	Unclear risk
Shalaby et al., 2024 [40]	Unclear risk	Unclear risk	Unclear risk	Unclear risk	Unclear risk	Unclear risk	Unclear risk	Low risk	Low risk	Unclear risk
Salih et al., 2018 [41]	Unclear risk	Unclear risk	Unclear risk	Unclear risk	Unclear risk	Unclear risk	Unclear risk	Low risk	Low risk	Unclear risk
Ugwuja et al., 2014 [42]	Unclear risk	Low risk	Unclear risk	Unclear risk	Unclear risk	Unclear risk	Unclear risk	Low risk	Low risk	Unclear risk

Table S1. Risk of bias assessment of the included animal studies using the SYRCLE’s tool
